# Solution-Processed Transparent Conducting Electrodes for Flexible Organic Solar Cells with 16.61% Efficiency

**DOI:** 10.1007/s40820-020-00566-3

**Published:** 2021-01-04

**Authors:** Juanyong Wan, Yonggao Xia, Junfeng Fang, Zhiguo Zhang, Bingang Xu, Jinzhao Wang, Ling Ai, Weijie Song, Kwun Nam Hui, Xi Fan, Yongfang Li

**Affiliations:** 1 Ningbo Institute of Materials Technology and Engineering, Chinese Academy of Sciences, Ningbo, 315201 People’s Republic of China; 2 School of Physics and Electronics Science, Engineering Research Center of Nanophotonics and Advanced Instrument, Ministry of Education, East China Normal University, Shanghai, 200241 People’s Republic of China; 3 Beijing National Laboratory for Molecular Sciences, CAS Key Laboratory of Organic Solids, Institution of Chemistry, Chinese Academy of Sciences, Beijing, 100190 People’s Republic of China; 4 Nanotechnology Center, Institute of Textiles and Clothing, The Hong Kong Polytechnic University, Hung Hom, Kowloon, Hong Kong, 999077 People’s Republic of China; 5 Department of Material Science and Engineering, Hubei University, Wuhan, 430062 People’s Republic of China; 6 State Key Laboratory of Organic/Inorganic Composites, Beijing Advanced Innovation Center for Soft Matter Science and Engineering, Beijing University of Chemical Technology, Beijing, 100029 People’s Republic of China; 7 Joint Key Laboratory of the Ministry of Education, Institute of Applied Physics and Materials Engineering, University of Macau, Avenida da Universidade, Taipa, Macau SAR, 999078 People’s Republic of China

**Keywords:** Solution-processed transparent conducting electrode, Flexible organic solar cell, PEDOT:PSS, Trifluoromethanesulfonic acid doping, Solution processing

## Abstract

Nonfullerene organic solar cells (OSCs) have achieved breakthrough with pushing the efficiency exceeding 17%. While this shed light on OSC commercialization, high-performance flexible OSCs should be pursued through solution manufacturing. Herein, we report a solution-processed flexible OSC based on a transparent conducting PEDOT:PSS anode doped with trifluoromethanesulfonic acid (CF_3_SO_3_H). Through a low-concentration and low-temperature CF_3_SO_3_H doping, the conducting polymer anodes exhibited a main sheet resistance of 35 Ω sq^−1^ (minimum value: 32 Ω sq^−1^), a raised work function (≈ 5.0 eV), a superior wettability, and a high electrical stability. The high work function minimized the energy level mismatch among the anodes, hole-transporting layers and electron-donors of the active layers, thereby leading to an enhanced carrier extraction. The solution-processed flexible OSCs yielded a record-high efficiency of 16.41% (maximum value: 16.61%). Besides, the flexible OSCs afforded the 1000 cyclic bending tests at the radius of 1.5 mm and the long-time thermal treatments at 85 °C, demonstrating a high flexibility and a good thermal stability.

**Electronic supplementary material:**

The online version of this article (10.1007/s40820-020-00566-3) contains supplementary material, which is available to authorized users.

## Highlights

- The PEDOT:PSS flexible electrodes with a unique CF_3_SO_3_H treatment exhibited high electrical characteristics and stability.
- An energy level tuning effect was induced to create a suitable work function.
- Flexible organic solar cells yielded a record-high efficiency of 16.61%, a high flexibility, and a good thermal stability.

**Electronic supplementary material:**

The online version of this article (10.1007/s40820-020-00566-3) contains supplementary material, which is available to authorized users.

## Graphical abstract

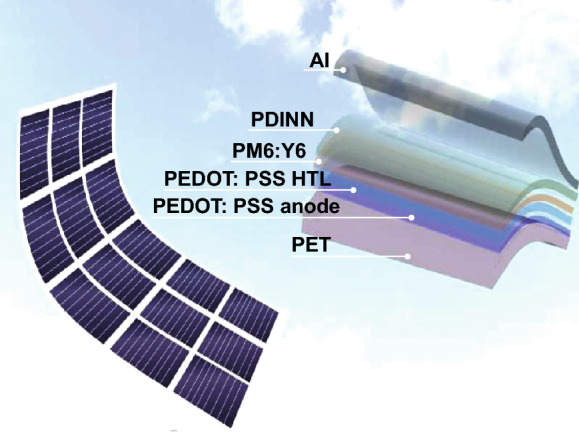

## Introduction

Flexible organic solar cells (OSCs) have become a popular research field, owing to the advantages of low cost, light weight, ease of fabrication, wearability, portability, etc. [1–16]. Transparent electrode fabrication is regarded as one of cores that determine the power conversion efficiency (PCE) and the device fabrication cost [17]. A flexible OSC requires that transparent electrodes on plastic substrates can exhibit high conductivity, high transparency, and good mechanical flexibility. However, the traditional transparent electrodes of indium tin oxide (ITO) have a low conductivity on plastic substrates and the ITO electrodes are easy to crack sharply in bending tests [18–21], making them unsuitable for application in flexible optoelectronics.

Several emerging transparent conducting materials show a promise as ITO alternatives and have the potential for large-area coverage and some degrees of mechanical flexibility. These materials include transparent conducting polymers [22–27], carbon nanotubes [28], graphene [14, 29], and metallic nanowires [2, 5, 6]. Among them, the solution-processed conducting polymeric films of poly(3,4-ethylenedioxythiophene):poly(4-styrenesulfonate) (PEDOT:PSS) were under intense investigation. The PEDOT:PSS films were widely used as the transparent electrode in OSCs, perovskite solar cells, light emitting diodes, etc. [17, 21]. Recent effort was devoted to the chemical doping of PEDOT:PSS films for improving the electrical characteristics. Doping strategies for the transparent PEDOT:PSS electrodes can be generally classified into a few categories: (i) metal chlorides such as FeCl_3_ and AuCl_3_ [14]; (ii) secondary polar solvents [30–32]; (iii) fluorosurfactant and Triton X-100 [33]; (iv) ionic liquids [34–36]; and (v) strong and mild acids [24–27, 37–40]. After the metal chloride and polar solvent doping, the PEDOT:PSS films exhibited a moderate electrical conductivity of 500–1000 S cm^−1^. Whereas, for the ionic liquid and fluorosurfactant doping, large-domain aggregates (> 0.40 μm) were generated in PEDOT:PSS aqueous solutions due to screening effects [12, 41]. Although acid post-treatments avoided the generation of large-domain aggregates, both high-temperature acid treatments (> 140 °C) and room-temperature 98 wt% H_2_SO_4_ treatments destroyed most of underlying plastic substrates [15, 25, 37, 40]. Therefore, it is challenging to prepare a highly conductive, smooth and flexible PEDOT:PSS electrode required by flexible OSCs.

Trifluoromethanesulfonic acid (CF_3_SO_3_H) is a super acid with ultrahigh acidity (*p*Ka = −15) over sulfuric acid (H_2_SO_4_, *p*Ka = −3.0) and methanesulfonic acid (CH_3_SO_3_H, *p*Ka = −1.9); thereby it provides a strong protonation of hydrogen ions (H^+^) to insulating PSS for raising the film conductivity. Moreover, owing to a polarization of carbon–fluorine (C–F) polar covalent bonds of CF_3_SO_3_H with uncoupling charge centers, CF_3_SO_3_H is capable of polarizing the PEDOTs and raising the work function of the PEDOT:PSS films. It potentially minimizes the energy level mismatch among the PEDOT:PSS electrodes, PEDOT:PSS (Clevios P VP AI4083) buffer layers, and electron-donors of active blends for an effective charge transport. It should be noted that, although 98 wt% CF_3_SO_3_H treatment was mentioned before, the harsh treatment made the PEDOT:PSS films unsuitable for application into flexible optoelctronics, because underlying plastic substrates were destroyed in the requisite strong acid process (Fig. S1). Besides, a combined pre-treatment using CF_3_SO_3_H and ionic liquids was developed for making transistor fabrications [12]. However, acid pre-treatments caused large aggregates in PEDOT:PSS aqueous solutions, which were unfavorable for the smooth and uniform anodes required by flexible OSCs. Thus, it is necessary and urgent to develop a unique CF_3_SO_3_H treatment for the development of the solution-processed PEDOT:PSS electrodes for flexible OSCs.

In this work, we proposed a low-temperature and low-concentration CF_3_SO_3_H post-treatment and demonstrated an efficient solution-processed flexible OSC based on the CH_3_SO_3_H-doped PEDOT:PSS films as transparent anodes and PM6:Y6 as active layers. The 0.8 M CF_3_SO_3_H post-treatment at 50 °C induced a series of merits, including low sheet resistances, high work functions, superior hydrophilicities and a little acid residue, for the PEDOT:PSS anodes. The optimized flexible OSCs exhibited an efficiency of 16.41% with the maximum value of 16.61%. To the best of our knowledge, 16.61% is the highest PCE for single-junction flexible OSCs reported so far. Furthermore, the solution-processed devices maintained a high flexibility and a good thermal stability. This work demonstrates the advance of the unique CH_3_SO_3_H post-treatment, and it provides a simple route to enable a flexible PEDOT:PSS anode with high conductivity, high work function and good stability for the realization of efficient and stable solution-processed flexible OSCs.

## Experimental Section

### Materials

PM6 and Y6 were purchased from Solarmer Materials Inc, Beijing. PEDOT:PSS aqueous solutions (i.e., Clevios PH1000 and Clevios P VP AI4083) were purchased from Heraeus, Germany.

### Device Fabrication

For the rigid ITO-based solar cells, glass substrates (size: 2.0 × 2.0 cm^2^) were cleaned through using ultrasonic treatments in deionized (DI) water, acetone, and isopropyl alcohol (IPA), and then were processed in UV-ozone chambers for 10 min. For the flexible solar cells, the polyethylene terephthalate (PET) plastic substrates were cleaned by IPA followed by baking at 100 °C for 10 min. Next, PEDOT:PSS aqueous solutions (PH1000) were filtered through 0.45 µm syringe filter. The PH1000 solutions were spin-coated on the as-employed substrates at 3500 rpm followed by the drying at 80 °C for 15 min in the air atmosphere. The spin-coating of PH1000 and the drying treatment were conducted again. Subsequently, the super acid treatment was conducted via dipping the CF_3_SO_3_H solutions on the PEDOT:PSS surfaces with a controlled temperature. Upon the CF_3_SO_3_H treatments for 5 min, the acid residues were washed off by DI-water and IPA followed by a baking at 80 °C for 15 min. Then, a PEDOT:PSS hole-transporting layer (Clevios P VP AI4083) was spin-coated on the PEDOT:PSS electrodes at 3000 rpm for 30 s, and the hole-transporting layer was dried at 100 °C for 10 min. After that, the active layer blends of PM6:Y6 were dissolved in chloroform with 1-chloronaphthalene (0.5%, v/v) and were spin-coated at 3000 rpm for 30 s onto the PEDOT:PSS (P VP AI4083) films. Then, perylene-diimide (PDINN) in methanol (1.0 mg mL^–1^) was spin-coated on the surfaces of active layers at 2000 rpm to obtain the electron transporting layers. Finally, the Al cathodes were thermally evaporated under a pressure at 10^–4^ Pa. The active area is 6.0 mm^2^. Note that metal probes were contacted with the Al top cathodes and the PEDOT:PSS bottom anodes coated with Ag pastes for the current density–voltage (*J*–*V*) characteristics.

### Characterizations

Sheet resistance was measured through using van der Pauw four-point probe method. Film thickness was conducted by a surface profile-meter (Talysurf Series II). The carrier concentration of the PEDOT:PSS electrodes was measured by the Hall measurement system (Lake Shore, 7704A) with the van der Pauw four-point probe method. UV–Vis spectra were taken on GS54T spectrophotometers (Shanghai Lengguang Technology Co., China). Film morphology was conducted through using a scanning probe microscope (SPM, VEECO Dimension 3100V) and transmission electron microscopy (TEM, TECNAI G20, FEI). The molecule structures were conducted through using Fourier transform infrared (FTIR) spectroscopy (NICOLET 6700, THermo, USA) and Raman spectroscopy (Renishaw inVia Reflex). Element compositions of the PEDOT:PSS films were conducted by X-ray photoelectron spectroscopy (XPS, XSAM800). Ultraviolet photoelectron spectra (UPS) measurements were carried out using a Kratos AXIS ULTRA DALD UPS system. *J*–*V* characteristics were measured in N_2_-filled glove-boxes using a Keithley 2400 sourcemeter under the illumination of AM 1.5G, with a AAA solar simulator (Newport, model 94023A). The lamp was calibrated by a 2 × 2 cm^2^ monocrystalline silicon reference cell (KG5 filter) provided by Newport Corporation. The light intensity was calibrated with a standard silicon detector. External quantum efficiency was conducted through the Newport quantum efficiency measurement system (ORIEL IQE 200TM) in the ambient atmosphere. The light intensity was calibrated with a standard Si/Ge solar cell. To evaluate the device flexibility, the solar cells were bent with radii of 1.5 mm and underwent the 1000 cycle harsh bending. To evaluate the thermal stability, the solar cells were placed in a hot plate at 85 °C in the glove-boxes filled with N_2_.

## Results and Discussion

### Optical and Electrical Characteristics of PEDOT:PSS Electrodes

**Fig. 1:**
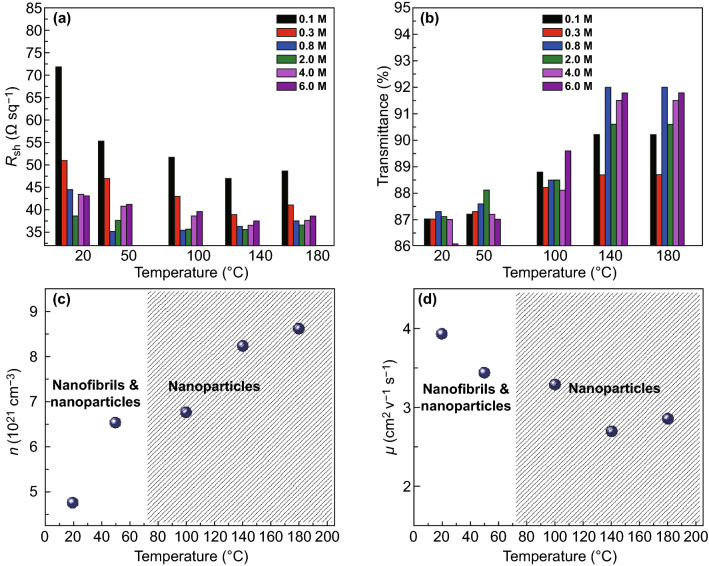
Electrical and optical characteristics of PEDOT:PSS anodes. **a** *R*_sh_ and **b** *T%* at *λ* = 550 nm of the PEDOT:PSS films with the different CF_3_SO_3_H doping treatments. **c** *n* and **d** *μ* of the PEDOT:PSS films with the 0.8 M CF_3_SO_3_H doping treatment at a variety of processing temperatures

Figure 1a, b plots the main sheet resistance (*R*_sh_) and the optical transparency (*T*%) at *λ* = 550 nm of the PEDOT:PSS electrodes with the CF_3_SO_3_H doping treatments. Except the PEDOT:PSS electrode with the 0.1 M CF_3_SO_3_H doping treatment at room temperature (RT: ≈ 20 °C) having *R*_sh_ of *≈* 72 Ω sq^−1^, all the films showed a low *R*_sh_ of 35–55 Ω sq^−1^. We found that with the increase in the doping treatment temperatures from RT to 140 °C, the *R*_sh_ of the PEDOT:PSS electrode was generally decreased. When the doping treatment temperatures were further raised from 140 to 180 °C, the *R*_sh_ was slightly increased for the PEDOT:PSS electrodes with 0.3–6.0 M CF_3_SO_3_H treatments. Through using the 0.8 M CF_3_SO_3_H treatment at 50 °C, the minimum sheet resistance of the optimized PEDOT:PSS electrode reached 32 Ω sq^−1^, which was attributed to: (i) a strong protonation of hydrogen ions that ionized from the super acids to the insulating components of PSS, and (ii) a polarization of fluorocarbon bonds to the PEDOT molecules.

The PEDOT:PSS electrodes showed a high optical transparency that was raised with the increase in the doping treatment temperatures. For example, after the 0.8 M CF_3_SO_3_H doping treatment at 180 °C, *T*% was increased from 87.3 to 91.7% at *λ* = 550 nm, which was accompanied with a morphology evolution and a reduced content of insulating PSS. With the lower concentration (0.1–0.8 M) CF_3_SO_3_H doping treatments at no higher than 50 °C, the PEDOT:PSS electrodes exhibited high optical transparencies over 87% in the visible regions of 400–550 nm (Fig. S2), which might be attributed to a large removal of hydrophilic PSS components from the PEDOT:PSS matrices as well as the formation of orderly stacked PEDOT molecules. The films with the 0.8 M CF_3_SO_3_H treatment at 50 °C having high optical and electrical characteristics should be suitable to be used as transparent electrodes for flexible OSCs.

We measured the carrier concentration (*n*) of the doped PEDOT:PSS films using the Hall effect. The carrier mobility (*µ*) of the PEDOT:PSS films was calculated from the relationship between the electrical conductivity (*σ*) and the carrier concentration: *σ* = *neμ*. Figure 1c, d plots the *n* and *µ* of the PEDOT:PSS films doped with 0.8 M CF_3_SO_3_H. With the increase in the doping treatment temperatures, *n* was increased from 4.77 to 8.62 cm^−3^, mostly attributed to the high protonation of the lower concentration CF_3_SO_3_H to PSS (H^+^ ionized from CF_3_SO_3_H) and polarization of C–F polar covalent bonds. A strong polarization induced weak Coulomb interactions between PEDOT and PSS, thereby leading to a favorable phase-separated morphology. The *n* values (6.52–8.62 cm^−3^) of the PEDOT:PSS films with 0.8 M CF_3_SO_3_H treatments are comparable to the best value (≈ 6.2 cm^−3^) of the optimal PEDOT:PSS films with 98 wt% sulfuric acid treatments [25]. Notably, the lower concentration CF_3_SO_3_H treatments avoided destroying underlying plastic substrates (Fig. S1), and it would reduce substantially strong acid residues on electrode surfaces. We also found that the *µ* values (3.93 and 3.44 cm^2^ V^−1^ s^−1^) of the PEDOT:PSS films doped by CF_3_SO_3_H at low temperatures (≤ 50 °C) were higher than that of the PEDOT:PSS films doped by CF_3_SO_3_H at high temperatures (≥ 100 °C). The higher *µ* values were related to a morphology evolution of these doped films. The results demonstrated a high electrical characteristic achieved by the PEDOT:PSS films with the CF_3_SO_3_H treatments; and the low-temperature and low-concentration CF_3_SO_3_H treatments are compatible to thermoplastic substrates for flexible device integration.

### Morphological and Structural Characteristics of PEDOT:PSS Electrodes

**Fig. 2:**
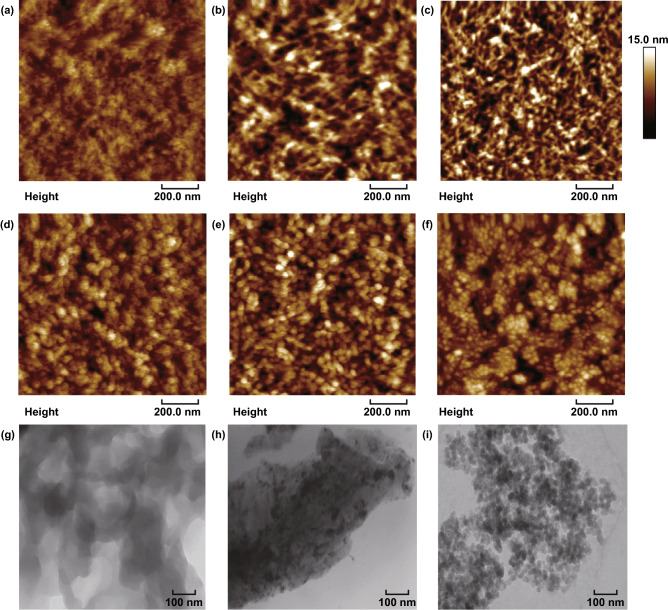
Morphological characteristics of PEDOT:PSS films. **a**, **g** As-cast films. The films with the 0.8 M CF_3_SO_3_H treatments at the temperatures: **b** RT; **c**, **h** 50 °C; **d** 100 °C; **e**, **i** 140 °C; and **f** 180 °C. Scale bar: 100 nm for **g**, **h,** and **i**

The high electrical characteristics are mostly attributed to an evolution in phase-segregated morphology and a reduced content of insulating PSS components. Figure 2 presents the morphologies of the PEDOT:PSS films (PH1000) through using SPM and TEM. As-cast films exhibited an inferior phase segregation with a low root-mean-square (RMS) roughness of 1.51 nm (Fig. 2a, g). Via the 0.8 M CF_3_SO_3_H doping at RT, the PEDOT:PSS films exhibited the physically continuous networks with a higher roughness of 2.63 nm. The morphology differs from the cluster morphology of the conventional PEDOT:PSS films that were doped with 6 vol% dimethylsulfoxide (DMSO) (Fig. S3). With the increase in the doping treatment temperatures to 50 °C, it induced a physically continuous network that consisted of spherical/elliptical-like nanoparticles and nanofibrils (Fig. 2c, h). The results demonstrated a favorable phase-segregated morphology, thanks to a full penetration of H+ into the PEDOT:PSS matrix followed by the reaction (H+ + PSS− → PSSH). The morphology was favorable for charge-carrier transport and collection by the PEDOT:PSS anodes in solar cells. With the CF_3_SO_3_H treatments at the higher temperatures of 100 and 140 °C, the PEDOT:PSS films showed a morphology evolution from linear/expended nanofibril-like networks to coiled small aggregated nanoparticles (Fig. 2d, e, g). The major sizes of the small nanoparticles were 20–40 nm. By increasing the doping treatment temperatures to 180 °C, the smallest nanoparticles with major sizes of 10–25 nm appeared (Fig. 2f, i), suggesting a weak Columbic attraction between the PEDOT molecules and the PSS chains. The PEDOT:PSS (PH1000) films are then characterized by the FTIR spectroscopy. As shown in Fig. S4a, the FTIR bands located between 1000 and 1100 cm^−1^ originate from the stretching vibrations of the PSS–H bonds [41, 42]. After the CF_3_SO_3_H treatments, the valleys blue-shifted and the valley’s intensity became stronger, indicating the PSSH formations in the PEDOT:PSS matrices. Figure S4b shows the Raman spectra of the PEDOT:PSS (PH1000) films including the as-cast ones, and the as-doped ones with the 0.8 M CF_3_SO_3_H treatments at 50 and 140 °C. The peak at 756 cm^−1^ corresponds to the stretching mode of CF_3_SO_3_^−^ [43]. The peak at 756 cm^−1^ wasn’t observed in the films, suggesting little CF_3_SO_3_H residuals on the surfaces of the CF_3_SO_3_H-doped films. The strongest band between 1400 and 1500 cm^−1^ originated from the C_α_=C_β_ stretching vibration of the PEDOTs. The C_α_=C_β_ vibration peaks in Raman spectroscopy red-shifted and were narrower in width in the as-doped PEDOT:PSS films as compared to that of the as-cast films. The results indicate an evolution of the PEDOTs from benzoid structures to quinoid structures, leading to a more planar backbone. This planarity is probably attributed to more efficient charge delocalization and a higher packing order [44]. The peak at 700 cm^−1^ corresponds to the sym C–S–C vibrations in PEDOTs [45]. The peak had a weak intensity and became wider in width, probably attributed to the formation of the quinoid structures of the PEDOTs.

### Components and Work Functions of PEDOT:PSS Electrodes

**Fig. 3:**
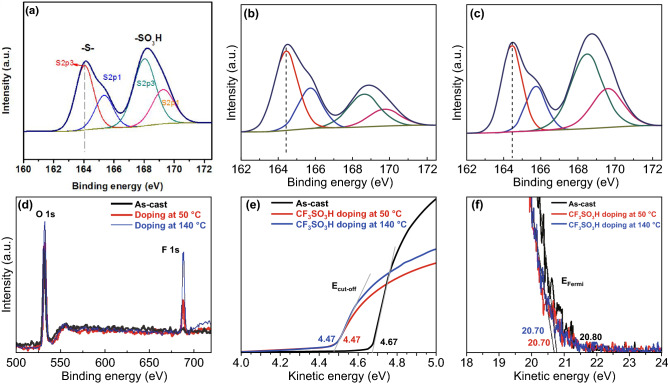
Element analysis and energy levels of PEDOT:PSS films. **a–c** Fitted S 2p XPS of the as-cast ones for **a** and both ones doped at 50 °C for **b** and 140 °C for **c**. **d** Fluorine contents of these films. UPS of the inelastic cutoff region for **e** and the HOMO region of the films for **f**

To elucidate the effect of the CF_3_SO_3_H doping treatments on the components of the PEDOT:PSS films, we showed the XPS full spectra (Fig. S5) and the fitted curves (Fig. 3a–c). For as-cast films, the ratio of sulfur (S) atoms in sulfonate moieties of PSS to S atoms in thiophene rings of PEDOT (called S_PSS_:S_PEDOT_) is 1.26:1 (Fig. 3a), whereas for the PEDOT:PSS films with the 0.8 M CF_3_SO_3_H treatment at 50 °C, the ratio is sharply decreased to 0.37:1 (Fig. 3b), indicating a large removal of insulating PSS from the PEDOT:PSS matrices. Note that, to accurately calculate the ratio, sulfonate moieties originated from CF_3_SO_3_H were considered as well. For the PEDOT:PSS films with the 0.8 M CF_3_SO_3_H treatment at 140 °C, the ratio is 0.82:1 (Fig. 3c). The less PSS components contributed to the reduced sheet resistances. Besides, the content of the fluorine atoms is 3.75 and 7.40 atom% for the PEDOT:PSS films doped at 50 and 140 °C, respectively (Fig. 3d). The electron-withdrawing fluorine groups led to the interfacial dipoles pointing toward the active layers of the OSCs. We also found that the CF_3_SO_3_H contents on the PEDOT:PSS film surfaces were much lower than that (14.08 atom%) of the PEDOT:PSS films with 40 wt% viscous phosphoric acid treatments [15], due to the use of the lower concentration CF_3_SO_3_H solutions with a low viscosity. It is favored to make a smooth anode and a high-quality coating of buffer layers on tops. Furthermore, we compared the binding energies of the S 2p_3/2_ emission for PEDOT to evaluate the impact of the doping treatments on the oxidation states of sulfurs of PEDOT. Binding energy of S 2p_3/2_ of PEDOT in as-cast films is 164.0 eV [39]. Both PEDOT:PSS films doped at 50 and 140 °C showed a visible shift of ≈ 0.34 eV toward higher binding energies of PEDOT emission, as compared to as-cast ones. The results demonstrated an evolution in the electronic environment of the sulfur atoms and a raised oxidation level of the thiophene sulfur of PEDOT molecules.

We probed the energy levels of the PEDOT:PSS films (PH1000) through UPS. Figure 3e, f shows the low kinetic energy cutoff (*E*_cutoff_) and the Fermi levels (*E*_Fermi_) of the PEDOT:PSS films including as-cast ones and 0.8 M CF_3_SO_3_H-doped ones. According to the relation below:

$$\phi = h\nu + E_{{{\text{cutoff}}}} - E_{{{\text{Fermi}}}}$$

Work function (*Φ*) is obtained. The CF_3_SO_3_H-doped films exhibited a high *Φ* of ≈ 5.0 eV (4.99 eV), which was independent of the doping treatment temperatures. It allowed a formation of Ohmic contacts, and it was favored for hole transport from active layers of OSCs to PEDOT:PSS anodes. Besides, the small offset between the *Φ* of PEDOT:PSS and the highest occupied molecular orbital (HOMO) of electron donors of active layers could maximize the charge extraction and minimize the recombination losses, thereby resulting in a raised built-in field for a high open-circuit voltage (*V*_OC_) of OSCs. These results encouraged us to apply the highly conductive PEDOT:PSS anodes with a high work function into efficient solution-processed flexible OSCs.

### Device Efficiency and Flexibility

**Fig. 4:**
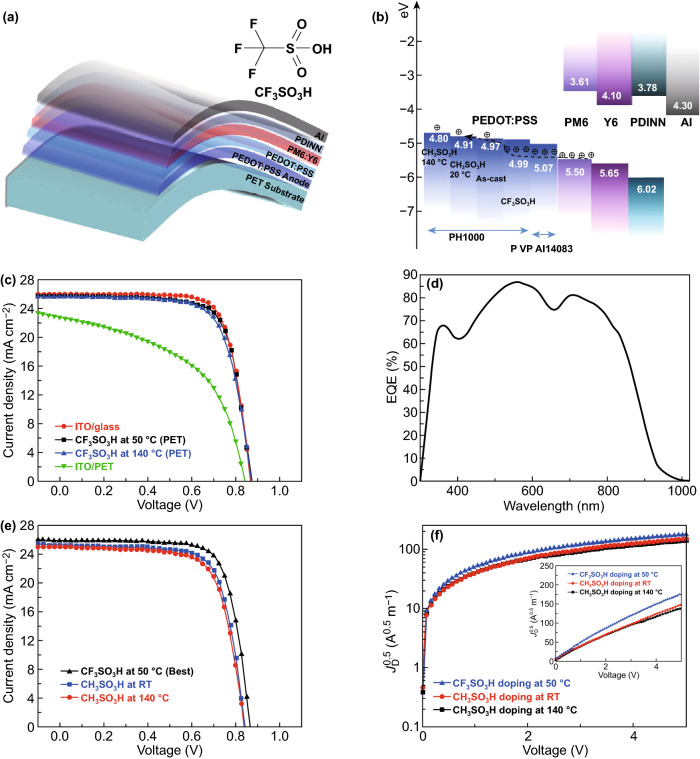
Flexible OSC characteristics. **a** Schematic diagrams. **b** Energy levels. **c** *J*–*V* characteristics of flexible OSCs with PEDOT:PSS and ITO anodes, and the OSCs fabricated on ITO/glass substrates. **d** EQE of flexible OSCs based on the PEDOT:PSS anodes with 0.8 M CF_3_SO_3_H treatments at 50 °C. **e** *J*–*V* characteristics of flexible OSCs with the CH_3_SO_3_H-doped anodes, and the best flexible OSC with the CF_3_SO_3_H-doped anodes. **f** Electric filed dependence of *J*_D_^0.5^ of the hole-only devices based on the anodes with the CF_3_SO_3_H and CH_3_SO_3_H treatments

We fabricated the ITO-free flexible OSCs based on the PEDOT:PSS anodes with the 0.8 M CF_3_SO_3_H doping treatment at the low temperature of 50 °C. Notably, most of plastic substrates suffered from a harsh strong acid treatment at high temperatures (e.g., > 140 °C), whereas the low-temperature and low-concentration CF_3_SO_3_H doping treatment avoided destroying the PET substrates, and thus it enabled the flexible PEDOT:PSS anodes. Figure 4a displays the flexible OSC structure, that is, PET (100 μm)/CF_3_SO_3_H-doped PEDOT:PSS (Clevios PH1000, 75 nm)/PEDOT:PSS (Clevios P VP AI4083, 30 nm)/PM6:Y6 [46] (150 nm)/PDINN [4] (10 nm)/Al (100 nm). The energy levels of the device components are illustrated in Fig. 4b. We also plotted the work functions of the PH1000 films including as-cast ones, the conventional ones [26, 37] soaked with 99.5 wt% CH_3_SO_3_H at RT, and 8.0 M CH_3_SO_3_H at high temperatures (140 °C). The good matching in energy levels among the CF_3_SO_3_H-doped PEDOT:PSS anodes, PEDOT:PSS (P VP AI4083) buffer layers and PM6 allowed an efficient hole transfer into the polymeric anodes. Figure 4c shows the *J*–*V* curves of the flexible OSCs based on the PEDOT:PSS anodes with the CF_3_SO_3_H doping at 50 and 140 °C, the flexible OSCs based on a commercial ITO (≈ 180 nm)/PET substrate, and the rigid OSCs fabricated on ITO (≈ 110 nm)/glass substrates. Through using the CF_3_SO_3_H treatment at 50 °C, the flexible devices yielded a main PCE of 16.41% with a fill factor (FF) of 74.0% and a short-circuit current density (*J*_SC_) of 25.79 mA cm^−2^ under the illumination of AM 1.5G, 100 mW cm^−2^. The PCE was higher than that (PCE:15.97%) of flexible OSCs based on the anodes with 0.8 M CF3SO3H treatments at 140 °C, and that (PCE: 9.69%) of the ITO-based flexible OSCs, which was mostly attributed to the gentle 0.8 M CF3SO3H treatment at 50 °C that not only avoided destroying the underlying plastic substrates, but also induced the high conductivity and high work function for the PEDOT:PSS anodes. The PCE (16.41%) was comparable to 16.96% efficiency of the control devices fabricated on ITO (≈ 110 nm)/glass substrates that exhibited a FF of 76.0% and a *J*_SC_ of 25.97 mA cm^−2^. Figure 4d shows the external quantum efficiency (EQE) of the flexible OSCs based on the PEDOT:PSS anodes with the 0.8 M CF_3_SO_3_H treatment at 50 °C. The maximum EQE value reached 86.9% at *λ* = 560 nm and 80.8% at *λ* = 710 nm, which demonstrated the high *J*_SC_ of 25.79 mA cm^−2^. Besides, the EQE spectra of the control rigid OSCs with the 110-nm-thick ITO electrodes and the flexible OSCs with the 180-nm-thick ITO electrodes are shown in Fig. S6 for reference. These results suggest that the low-temperature (50 °C) and low-concentration (0.8 M) CF_3_SO_3_H treatment is suitable to prepare flexible PEDOT:PSS anodes that are promising for efficient solution-processed flexible OSCs.

To further present the advance of the CF_3_SO_3_H-doped PEDOT:PSS anodes, we fabricated the flexible OSCs based on PEDOT:PSS anodes with CH_3_SO_3_H treatments. As mentioned in the literature [15, 26, 37], the PEDOT:PSS anodes were prepared via using 99.5 wt% CH_3_SO_3_H treatments at room temperature [15, 37], and 8.0 M CH_3_SO_3_H treatments at 140 °C [26], respectively. As shown in Fig. 4e, the flexible OSCs showed an efficiency of 15.36% with a *V*_OC_ of 0.84 V, a *J*_SC_ of 25.22 mA cm^−2^ and a FF of 72.5% for the 8.0 M CH_3_SO_3_H treatments at 140 °C, and 14.79% with a *V*_OC_ of 0.84 V, a *J*_SC_ of 24.97 mA cm^−2^, and a *FF* of 70.5% for the 99.5 wt% CH_3_SO_3_H treatments at RT. The PCE values were much lower than that (the maximum PCE: 16.61%) of the best flexible OSCs with the PEDOT:PSS anode by the 0.8 M CF_3_SO_3_H treatment at 50 °C, the *J*–*V* curve of which is also shown in Fig. 4e. Table 1 summarizes the performance data of the OSC devices for reference.

**Table 1:** Photovoltaic performance of the OSCs based on PEDOT:PSS and ITO electrodes

Electrode	Doping and fabrication method	*V*_OC_ (V)	*J*_SC_ (mA cm^−2^)	FF (%)	PCE (%)
PEDOT:PSS/PET	0.8 M CF_3_SO_3_H doping at 50 °C	0.86	25.79	74.0	16.41 (± 0.20)
PEDOT:PSS/PET	0.8 M CF_3_SO_3_H doping at 50 °C	0.86	25.85	74.8	16.61 (Best)
PEDOT:PSS/PET	0.8 M CF_3_SO_3_H doping at 140 °C	0.86	25.68	72.3	15.97 (± 0.26)
ITO (180 nm)/PET	Magnetron sputtering	0.84	22.74	50.7	9.69 (± 0.41)
ITO (110 nm)/glass	Magnetron sputtering	0.84	25.97	76.0	16.96 (± 0.13)
PEDOT:PSS/PET	8.0 M CH_3_SO_3_H doping at 140 °C	0.84	25.22	72.5	15.36 (± 0.31)
PEDOT:PSS /PET	99.5 wt% CH_3_SO_3_H doping at RT	0.84	24.97	70.5	14.79 (± 0.26)

Over ten devices were reproduced to confirm the mean PCE

The efficiency is significantly promoted through using the optimized PEDOT:PSS (PH1000) anodes that affected the hole mobility (*μ*_h_) of devices. The *μ*_h_ values of three-kinds of hole-only devices with a structure of PET/PEDOT:PSS anodes/PEDOT:PSS (P VP AI4083)/PM6:Y6/Au were calculated by the space charge limited current (SCLC) model using Theott–Gurney square law [47]:

$$J_{{\text{D}}} = \frac{9}{8}\varepsilon_{0} \varepsilon_{{\text{r}}} \mu \frac{{V^{2} }}{{L^{3} }}$$

where *ε*_r_ is the dielectric constant of active layer materials, *ε*_0_ is the permittivity of free space, *L* is the distance between the polymeric anode and the metal cathode (Au), which is equivalent to the thickness of active layers, and *J*_D_ is the dark current density. Figures 4f shows the voltage dependence of the *J*_D_^0.5^ of the hole-only devices. The *µ*_h_ is 1.71 × 10^−4^, 2.63 × 10^−4^, and 3.46 × 10^−4^ cm^2^ V^−1^ s^−1^ for the hole-only OSCs based on the PEDOT:PSS anodes with 8.0 M CH_3_SO_3_H treatments at 140 °C, 99.5 wt% CH_3_SO_3_H treatments at RT, and 0.8 M CF_3_SO_3_H treatments at 50 °C, respectively. The CF_3_SO_3_H-doped PEDOT:PSS anode exhibited the highest hole mobility, which facilitated charge-carrier transport and collection for raising the efficiency of flexible OSCs.

The device efficiency is probably affected by the interface contacts between the PEDOT:PSS (PH1000) anodes and the PEDOT:PSS (PVP AI4083) hole-transporting layers (HTLs). In contact angle measurements, as-cast PEDOT:PSS (PH1000) films and the PEDOT:PSS anodes with CH_3_SO_3_H treatments at 140 °C and RT exhibited a mean contact angle of 38.5°, 30.0° and 23.5°, respectively, whereas the PEDOT:PSS anodes with 0.8 M CF_3_SO_3_H treatments at 50 °C showed the smallest contact angle of 19.0° (Fig. S7). An intimate contact was formed between the PEDOT:PSS anodes with the CF_3_SO_3_H treatment at 50 °C and the PEDOT:PSS (PVP AI4083) HTLs, which was attributed to: (i) the intermolecular forces between rich C–F bonds of CF_3_SO_3_H and sulfonate groups of PEDOT:PSS (PVP AI4083), and (ii) the hydrogen bonds between CF_3_SO_3_H and the sulfonate groups. The high hydrophilicity of the CF_3_SO_3_H-doped anodes was favorable for the deposition of the PEDOT:PSS (PVP AI4083) HTLs for a high *µ*_h_ and PCE.

**Fig. 5:**
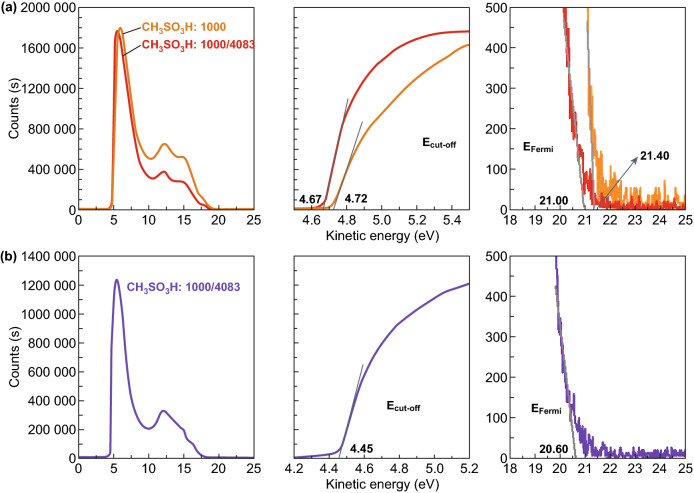
Work functions of the films. **a** UPS of the polymeric anodes with CH_3_SO_3_H treatments and the PEDOT:PSS (P VP AI4083)-coated anodes. **b** UPS of the CF_3_SO_3_H-doped anodes coated with PEDOT:PSS (P VP AI4083) buffer layers

We also observed that the 0.8 M CF_3_SO_3_H treatment at 50 °C induced the higher *Φ* of the anodes and the PEDOT:PSS (PVP AI4083)-coated anodes. As calculated from Fig. 5a, the *Φ* of the CH_3_SO_3_H-doped control anodes and the control anodes coated with PEDOT:PSS (PVP AI4083) is 4.54 and 4.89 eV, respectively. The difference (4.54 vs 4.99 eV) in *Φ* of both CH_3_SO_3_H- and CF_3_SO_3_H-doped PEDOT:PSS anodes is related to the polar natures of the acids employed. It suggests that fluorocarbon bonds of CF_3_SO_3_H provide a large dipole moment. When PEDOT:PSS (PVP AI4083) was coated on the optimized PEDOT:PSS anodes with the 0.8 M CF_3_SO_3_H treatment at 50 °C, the *Φ* was improved to be 5.07 eV (Fig. 5b). The match (4.99, 5.07 and 5.50 eV) in the energy levels of the CF_3_SO_3_H-doped PEDOT:PSS anode, HTL and PM6 allowed efficient hole transfer into the polymeric anodes. As a result, the efficiency of the solution-processed flexible OSCs was maximized.

**Fig. 6:**
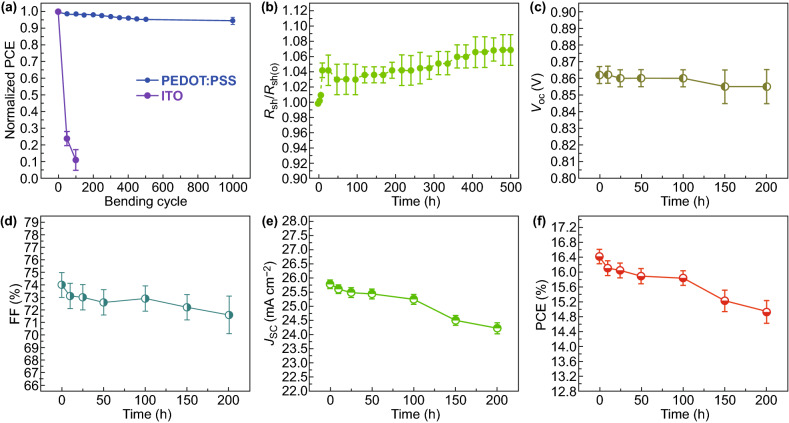
Mechanical property and thermal stability of the flexible OSCs. **a** Profiles of normalized PCE of flexible OSCs based on the CF_3_SO_3_H-doped anodes and ITO in harsh flexing tests at *r* of 1.50 mm. **b** *R*_sh_ versus the thermal processing time for the CF_3_SO_3_H-doped PEDOT:PSS anodes under 85 °C/85% relative humidity conditions. Profiles of photovoltaic parameters of the flexible OSCs based on the 0.8 M CF_3_SO_3_H-doped anodes as a function of the thermal processing time: **c** *V*_OC_, **d** FF, **e** *J*_SC_, and **f** PCE

The flexible OSCs have the advantage of high mechanical flexibility over the devices fabricated on ITO (180 nm)/PET substrates, as shown in Fig. 6a. With 1,000 cyclic bending at *r* of 1.5 mm, the flexible devices based on the CF_3_SO_3_H-doped PEDOT:PSS anodes maintained 94.4% of the initial efficiency, whereas the devices with the ITO (≈ 180 nm)/PET substrates showed a large drop in PCE down to 11.0% of the initial value at *r* of 1.5 mm, that meant, active regions of the ITO electrodes had been scrapped to restrain charge-carrier collections. We found that, for the small bending at the radius of 1.5 mm, the PEDOT:PSS anodes coated on the PET substrates (2 × 2 cm^2^; two edges were coated with Ag pastes) had no visible increase in resistance, while the resistance of the ITO/PET was increased to 3 orders of magnitude (from 1 to over 2,800), as shown in Fig. S8; this implies a structure damage of the ITO films with crack generations.

### Device Stability

Furthermore, we investigated the thermal stability of the PEDOT:PSS anodes and the as-integrated flexible OSCs. The PEDOT:PSS anodes were thermal annealed at 85 °C in the air atmosphere (relative humidity: 85%). As shown in Fig. 6b, the sheet resistance of the PEDOT:PSS anodes was increased by ≈ 3.6% for the 168 h testing and by ≈ 6.9% for 500 h testing. The PEDOT:PSS anodes had a slight increase in *R*_sh_ from 35.0 to 37.4 Ω sq^−1^ in the long-time thermal process for 500 h. A high thermal stability of the PEDOT:PSS anodes is energetically favored to raise the performance stability of the flexible OSCs. Figure 6c–f shows the photovoltaic parameters (*V*_OC_, FF, *J*_SC_, and PCE) of the flexible OSCs based on the optimized PEDOT:PSS anodes as a function of thermal processing time, respectively. Notably, the flexible OSCs were thermally processed at 85 °C for 200 h in an inert glove-box filled with nitrogen (N_2_). Obviously, *V*_OC_ is almost independent of the thermal processing times. The FF of the flexible devices changed a little, and it was of a high value (higher than 73.0%) for the flexible OSCs with the thermal treatment for 25 h. The FF of the flexible OSCs with the thermal treatment for 200 h is 71.6%, which was a little lower than that of the flexible OSCs in a shorter thermal process. However, the *J*_SC_ of the flexible OSCs changed significantly in the thermal process. As mentioned above, the flexible OSCs with the optimized PEDOT:PSS anodes showed the initial *J*_SC_ of 25.78 mA cm^−2^. Increasing the processing time to 150 and 200 h led to a lower *J*_SC_ of 24.51 and 24.23 mA cm^−1^, respectively. Thus, the PCE was decreased from the initial value of 16.41% to 14.92% in the thermal stability test. The decrease in PCE might be attributed to the insulating PSS components of the PEDOT:PSS anodes and P VPAI4083 buffer layers that absorbed moisture (e.g., alcohols, H_2_O), leading to a volume expansion. Besides, the rigid main-chain structures and irregular arrangement of PDINN molecules potentially caused a contact issue at interfaces and an electrical stability concern in the thermal process. The performance stability of solution-processed flexible devices would be promoted through removing PSS components from the PEDOT:PSS matrices, and employing stable buffer layers for an interface shield in the future.

## Conclusions

We demonstrated the flexible OSCs based on the 0.8 M CF_3_SO_3_H-doped PEDOT:PSS anodes and PM6:Y6 active layers. The flexible devices yielded the power conversion efficiency of 16.41% with the maximum value of 16.61%. It was mostly attributed to the low-temperature doping treatment using lower concentration CF_3_SO_3_H containing fluorine groups that endowed the PEDOT:PSS anodes with high optical and electrical characteristics and a higher work function (≈ 5.0 eV). The CF_3_SO_3_H-doped PEDOT:PSS anodes showed a favorable phase-separated morphology with physically continuous networks; and the films had a high charge concentration (6.54 × 10^21^ cm^−3^) and mobility (3.44 cm^2^ V^−1^ s^−1^), accounting for a low sheet resistance. The work function was raised by the electron-withdrawing fluorine groups of CF_3_SO_3_H that reduced the charge recombination loss at interfaces, substantially accounting for the improvement in *V*_OC_. Besides, the CF_3_SO_3_H-doped PEDOT:PSS anodes exhibited a better wettability, resulting in an intimate contact with the hole-transporting layers (P VP AI4083). The PCE of the solution-processed flexible OSCs was maintained well in the bending tests, in which 94.4% of the initial value was retained after 1000 cyclic bending at *r* of 1.5 mm. The solution-processed flexible OSCs also exhibited a good thermal stability in the 200 h thermal process at 85 °C, i.e., a decrease by 9.1% in PCE in the stability test. This work demonstrates a solution-processed flexible OSC with a high efficiency, a high flexibility and a good thermal stability. It widens the adaptation of the flexible PEDOT:PSS anodes into high-performance flexible optoelectronics.

## Electronic supplementary material

Below is the link to the electronic supplementary material.

Supplementary material 1 (PDF 442 kb)
