# Supplementary material for: Solution-Processed Transparent Conducting Electrodes for Flexible Organic Solar Cells with 16.61% Efficiency
Source: Nanomicro Lett. 2021 Jan 4;13:44. doi: 10.1007/s40820-020-00566-3 (PMC8187532; doi:10.1007/s40820-020-00566-3)
Supplement: Supplementary file 1 — Supplementary material 1 (PDF 442 kb) [file 40820_2020_566_MOESM1_ESM.pdf]

Supporting Information for

Solution-Processed Transparent Conducting Electrodes for Flexible Organic Solar Cells with 16.61% Efficiency

Juanyong Wan[1], Yonggao Xia[1], Junfeng Fang[2,*], Zhiguo Zhang[3,6,*], Bingang Xu[4], Jinzhao Wang[5], Ling Ai[1], Weijie Song[1], Kwun Nam Hui[7], Xi Fan[1,*], Yongfang Li[3]

[1]Ningbo Institute of Materials Technology and Engineering, Chinese Academy of Sciences, Ningbo 315201, P. R. China

[2]School of Physics and Electronics Science, Engineering Research Center of Nanophotonics & Advanced Instrument, Ministry of Education, East China Normal University, Shanghai 200241, P. R. China

[3]Beijing National Laboratory for Molecular Sciences, CAS Key Laboratory of Organic Solids, Institution of Chemistry, Chinese Academy of Sciences, Beijing 100190, P. R. China

[4]Nanotechnology Center, Institute of Textiles and Clothing, The Hong Kong Polytechnic University, Hung Hom, Kowloon, Hong Kong 999077, P. R. China

[5]Department of Material Science and Engineering, Hubei University, Wuhan 430062, P. R. China

[6]State Key Laboratory of Organic/Inorganic Composites, Beijing Advanced Innovation Center for Soft Matter Science and Engineering, Beijing University of Chemical Technology, Beijing 100029, P. R. China

[7]Joint Key Laboratory of the Ministry of Education, Institute of Applied Physics and Materials Engineering, University of Macau, Avenida da Universidade, Taipa, Macau SAR 999078, P. R. China

*Corresponding authors. E-mail: jffang@phy.ecnu.edu.cn (Junfeng Fang); zgzhangwhu@iccas.ac.cn (Zhiguo Zhang); fanxi@nimte.ac.cn (Xi Fan)

Supplementary Figures

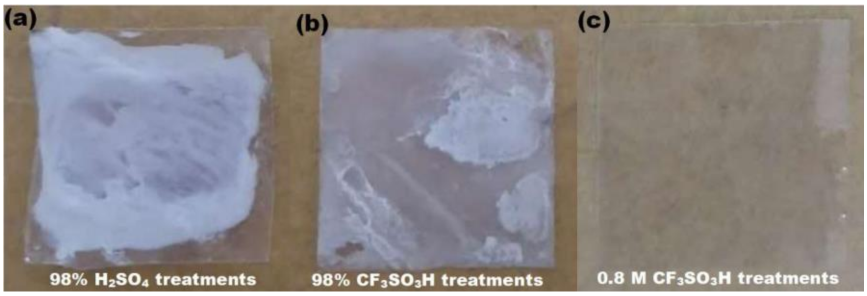

Fig. S1 Images of PET plastic substrates with (**a**) 98 wt% H_2SO_4 treatments at R.T. (**b**) 98 wt% CF_3SO_3H treatments at R.T. (**c**) low-concentration (0.8 M) CF_3SO_3H treatments at 50 °C. The underlying PET substrates were damaged by the 98 wt% H_2SO_4 and 98 wt% CF_3SO_3H treatments.

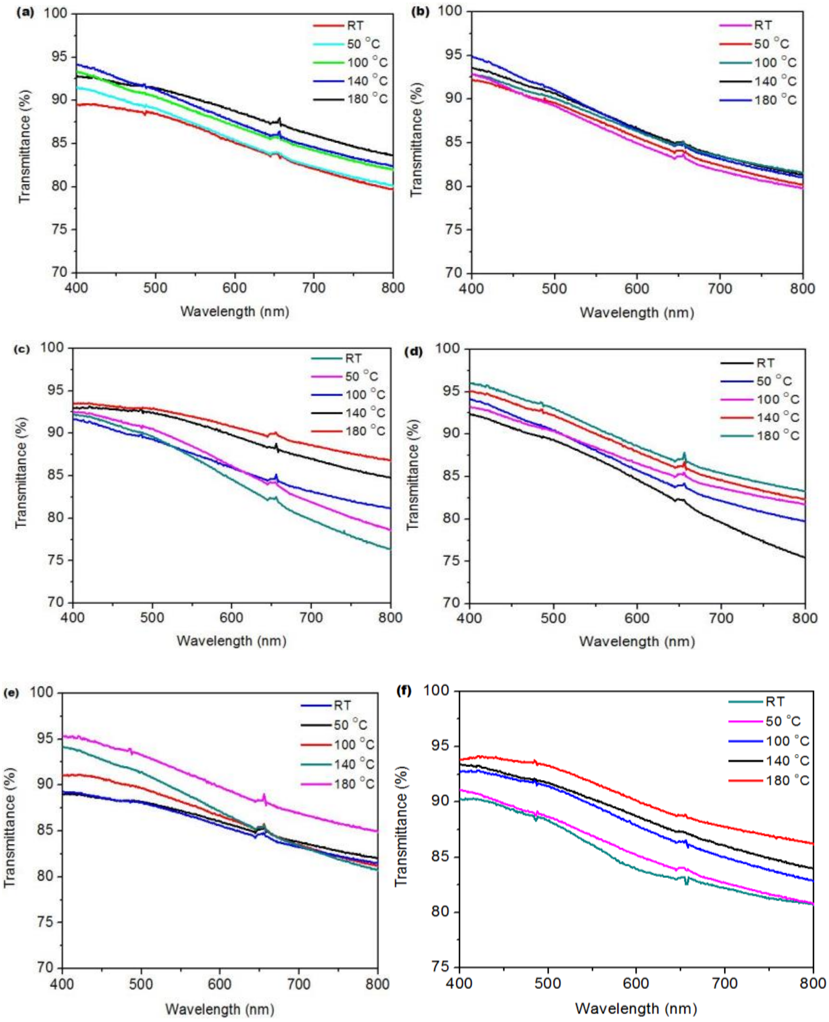

Fig. S2 Optical transparencies of the PEDOT:PSS films with the CF_3SO_3H treatments: (**a**) 0.1 M; (**b**) 0.3 M; (**c**) 0.8 M; (**d**) 2.0 M: (**e**) 4.0 M; and (**f**) 6.0 M

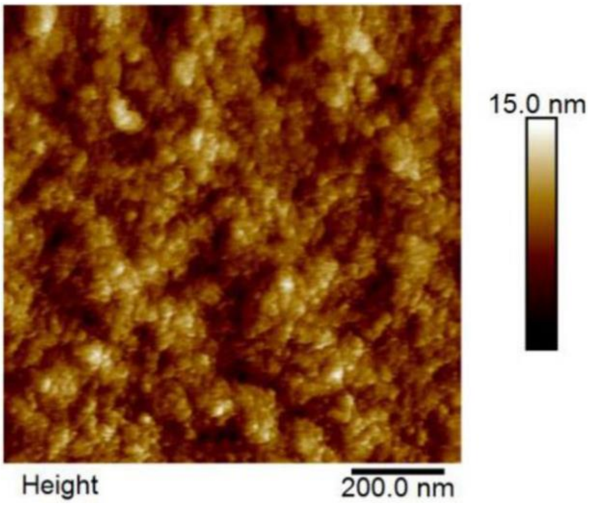

Fig. S3 Morphology of the PEDOT:PSS films with 6 vol% DMSO treatments

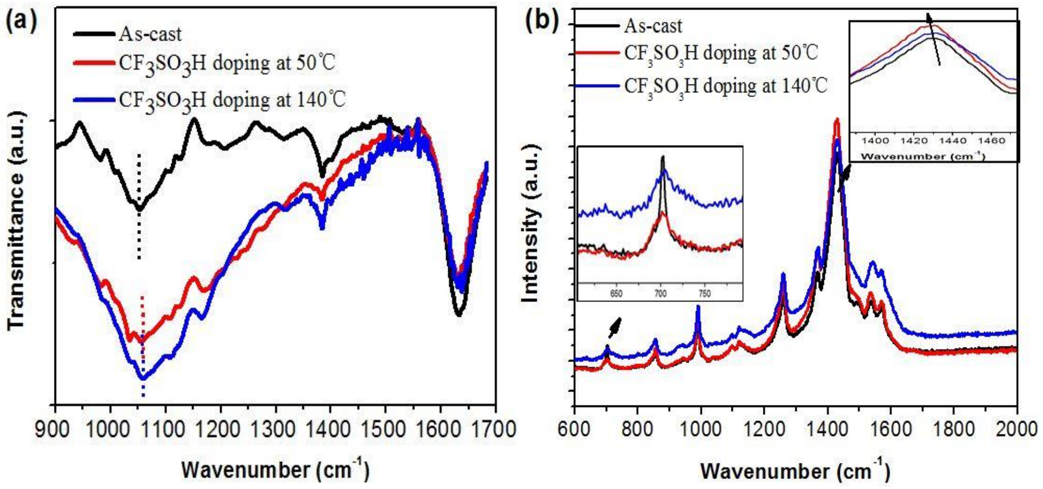

Fig. S4 FTIR (**a**) and Raman spectra (**b**) of the as-cast polymeric films and the PEDOT:PSS films with CF_3SO_3H doping treatments at 50 and 140 °C, respectively

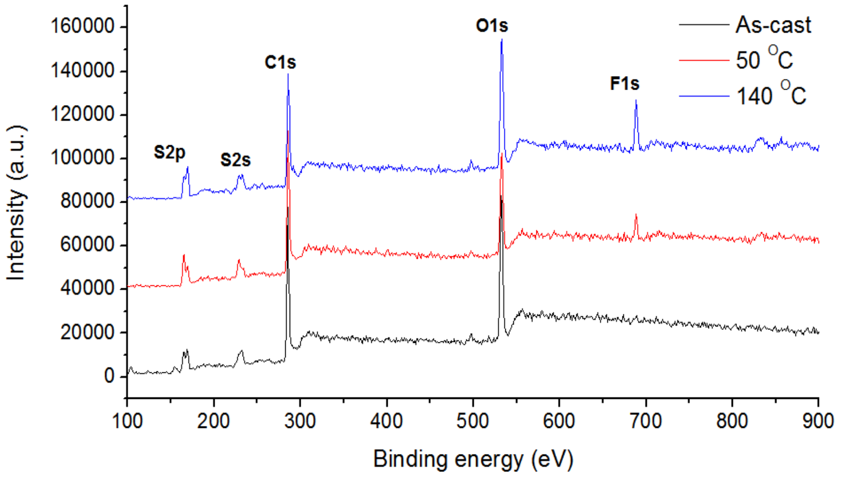

Fig. S5 XPS of the as-cast polymeric films and the PEDOT:PSS films with CF_3SO_3H doping treatments at 50 °C and 140 °C

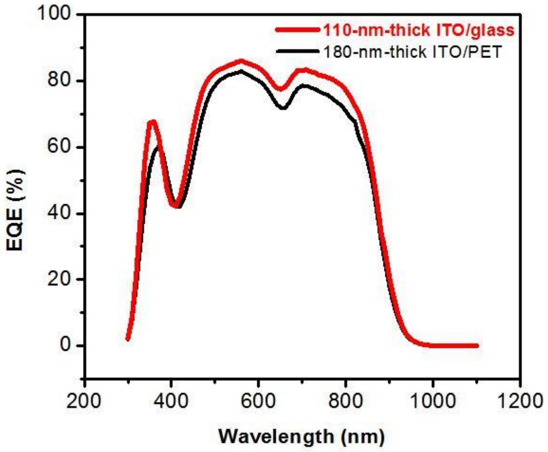

Fig. S6 EQE spectra of the control OSCs fabricated on the 110-nm-thick ITO electrodes on glass substrates and the flexible OSCs fabricated on the 180-nm-thick ITO electrodes on PET substrates

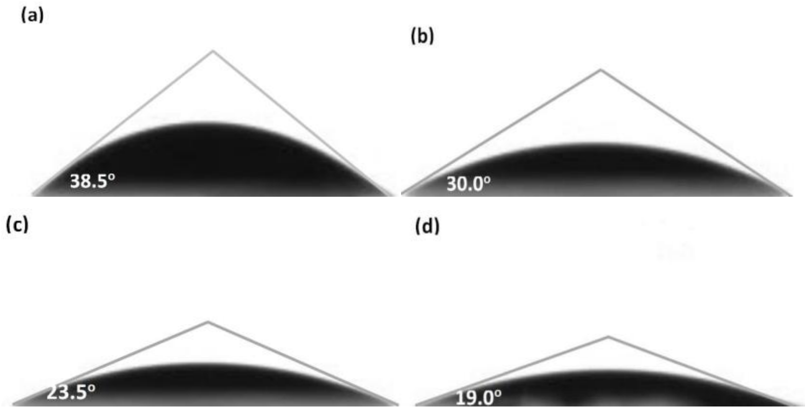

Fig. S7 Wettability characteristics of PEDOT:PSS (P VP AI4083) droplets on the surfaces of these films (PH1000): (**a**) as-cast; (**b**) 99.5 wt% CH_3SO_3H doing at 140 °C; (**c**) 8.0 M CH_3SO_3H doping at r.t.; and (**d**) 0.8 M CF_3SO_3H doping at 50 °C

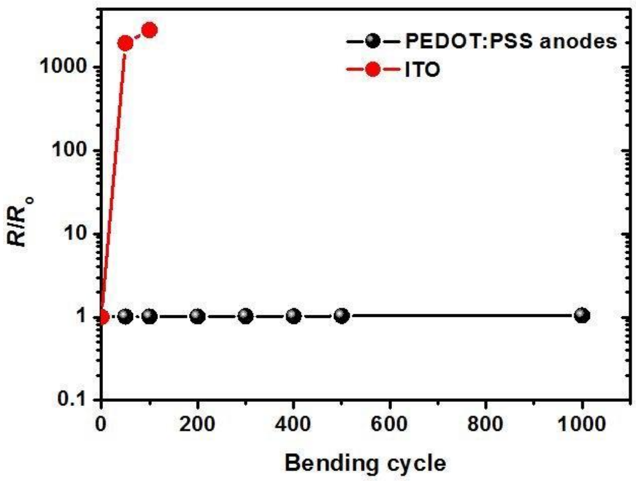

Fig. S8 R/R_o of the 0.8 M CF_3SO_3H-doped PEDOT:PSS (75 nm)/PET substrates and the (180 nm) ITO/PET substrates as a function of bending cycles at r of 1.5 mm
